# Supplementary material for: Normalization by valence and motivational intensity in the sensorimotor cortices (PMd, M1, and S1)
Source: Sci Rep. 2021 Dec 20;11:24221. doi: 10.1038/s41598-021-03200-3 (PMC8688489; doi:10.1038/s41598-021-03200-3)

Normalization by Valence and Motivational Intensity in the Sensorimotor Cortices (PMd, M1 and S1)

SUPPLEMENTARY INFORMATION

Zhao Yao[2*], John P Hessburg[2*] and Joseph Thachil Francis[1,3]

Correspondence: joey199us@gmail.com

Acknowledgments: Research was supported by NIH 1R01NS092894-01, NSF IIS-1527558.

[1] Department of Biomedical Engineering and Electrical and Computer Engineering, Cullen College of Engineering at The University of Houston, Houston, TX77204, United States;

[2] Department of Physiology and Pharmacology, State University of New York Downstate Medical Center, Brooklyn, NY11203, United States;

* Co-First Authorship. [3] Lead Contact - joey199us@gmail.com

The figure below shows the electrode arrays' placement in the two NHP subjects in the current study.

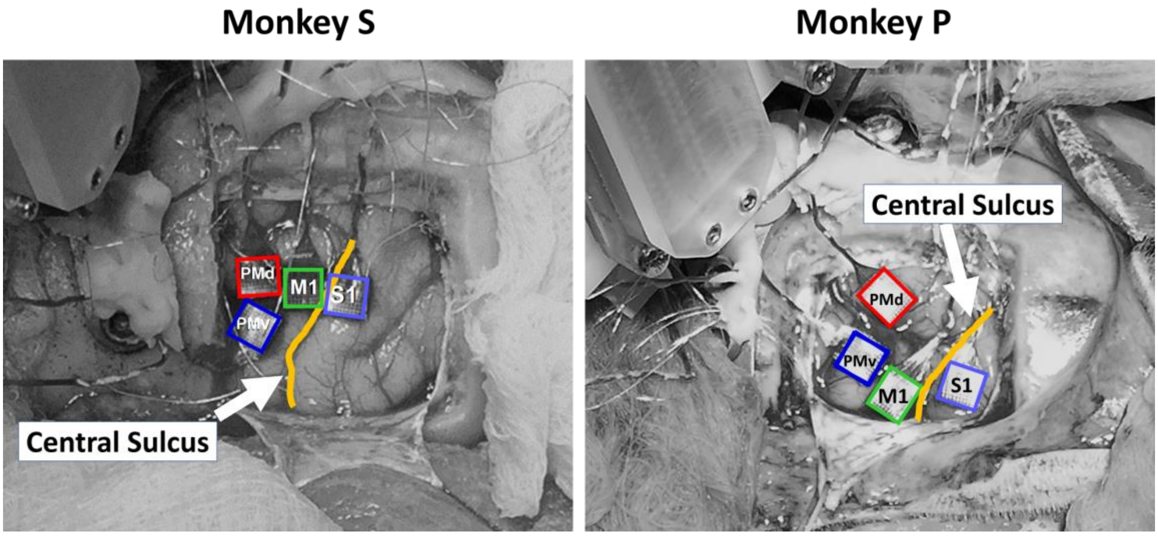

Fig.S.1: Position of four Utah arrays in relation to the central sulcus, yellow line, for NHP S (left) and P (right). The four arrays were implanted in caudal S1 (cS1), rostral M1 (rM1), PMd, and PMv cortices. Note that in NHP P, we had to implant the rM1 array more lateral than in NHP S due to a large set of blood vessels running through that region. Likewise, PMd was implanted more medial in NHP P for this exact reason compared to NHP S. PMv data was not utilized in this report due to noise issues.

Fig.S.2. Successive Statistical Tests Give Increasing Statistical Significance

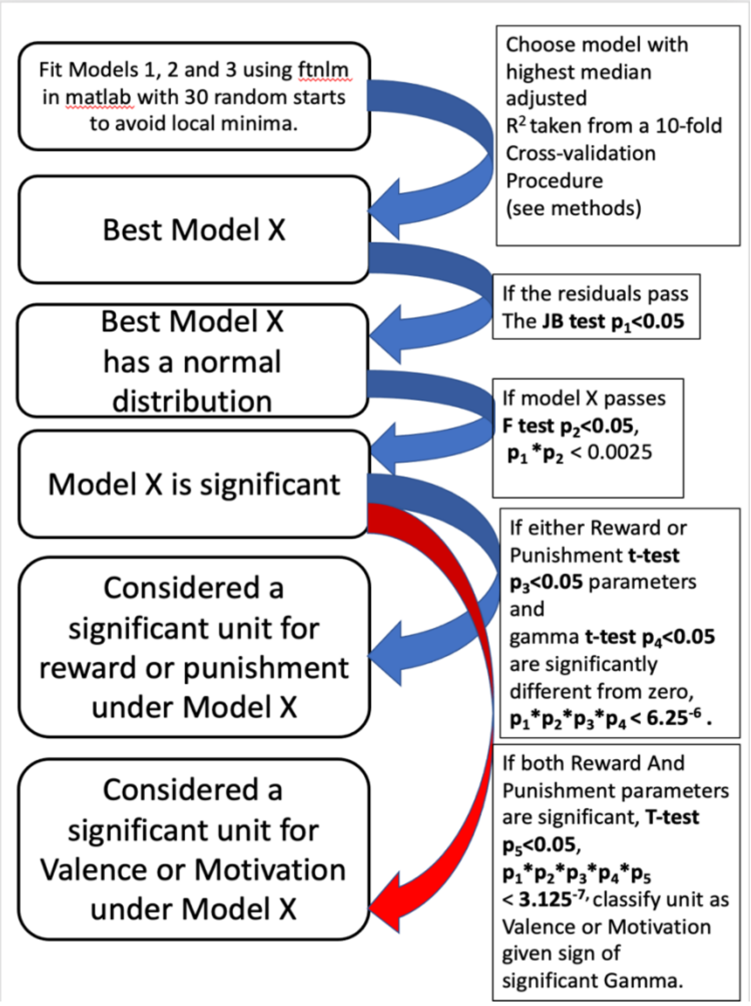

Fig.S.2. Our analysis divided the reward/ punishment modulation units into 3 groups: 1) Units only modulated by reward. 2) Units only modulated by punishment. 3) Units modulate by both reward and punishment. In group 3, we then analyzed if the units were valence or motivational (see figure.7b and 7c). As seen from Fig.S.2, we are not running these statistical tests in parallel but rather in succession. The associated p values given for the successive testing assume independence of tests and can be seen as the best-case p-value.

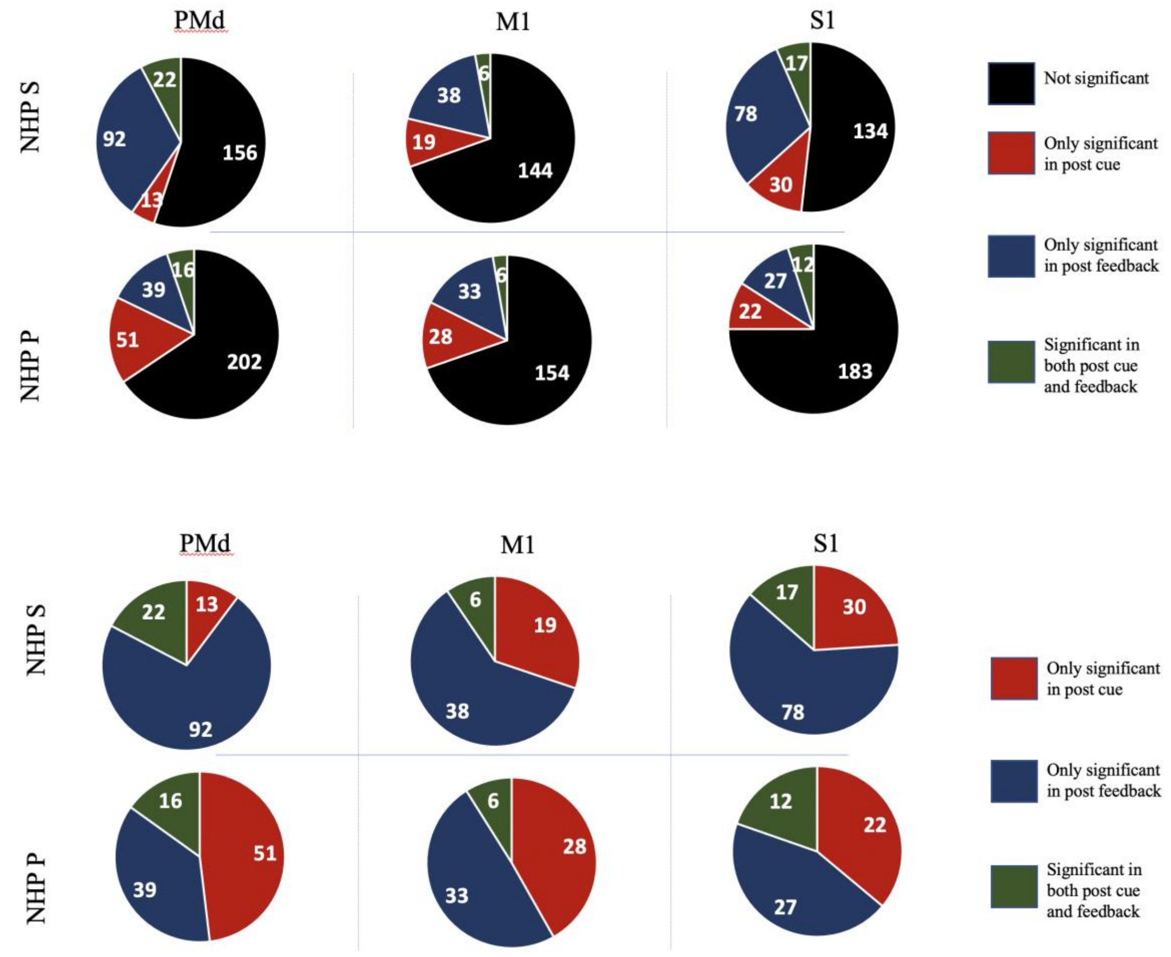

Fig.S.3: Here, we show the significant units and their makeup and the perspective of the larger non-significant population (Top subplot). For convenience, we have copied Fig.7 from the main text below it.

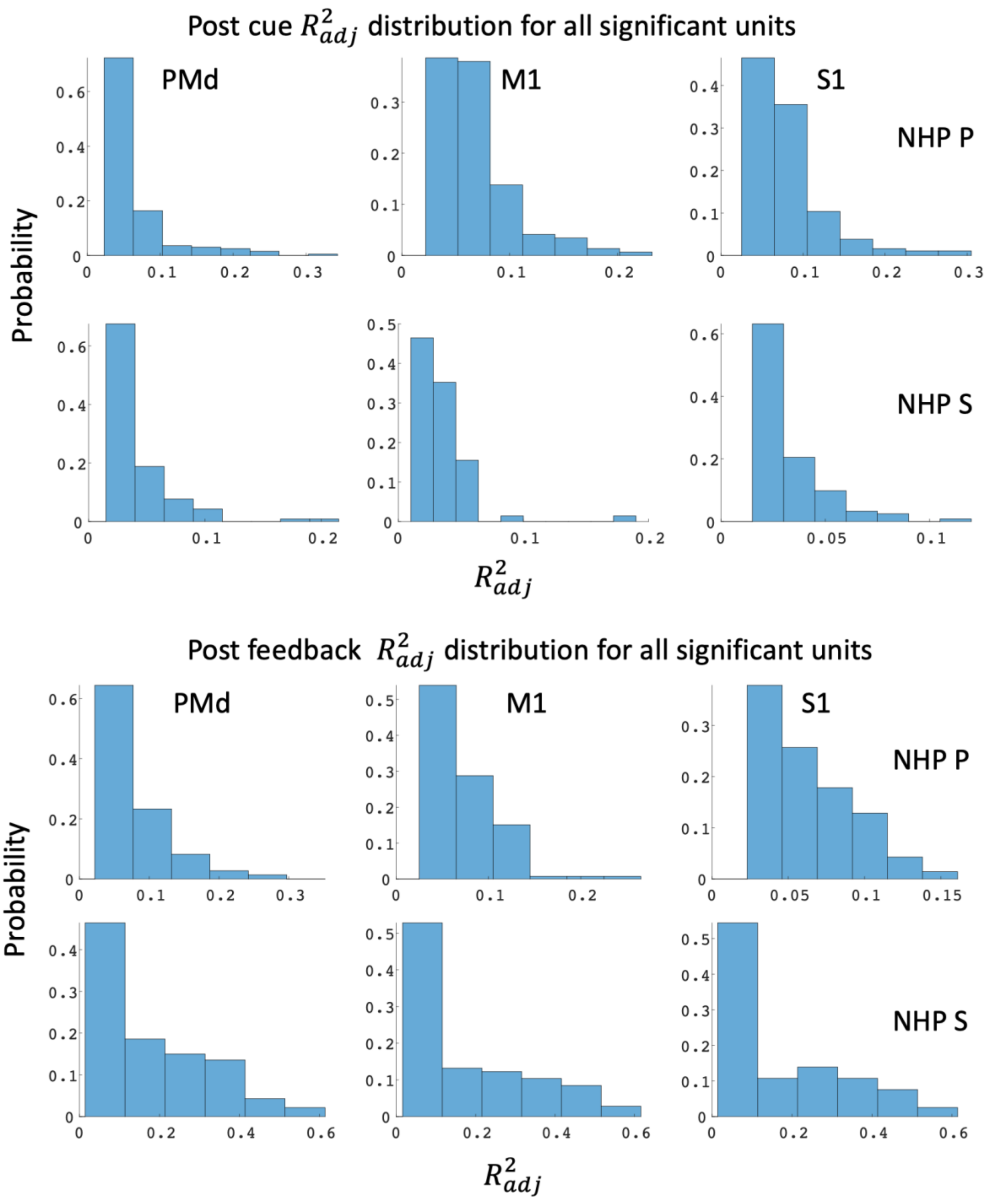

Fig.S.4. Distribution of the adjusted R squared values for the best significant model fit for a given unit during the post-cue, top two rows, and the post-feedback, bottom two rows, for the two NHPs labeled on the right-hand side of the figure. Fitting firing rates from every trial as

compared to Fig.9 in the main text that comes from the model fits to the mean for each of the 16 different affective stimuli.

Fig.S.5. showing only significant units as compared to all units as seen in the main text table.3. Often, but not always, model #2, which utilized the R and P levels from the task in the denominator, outperformed model #3, which used the local population activity.

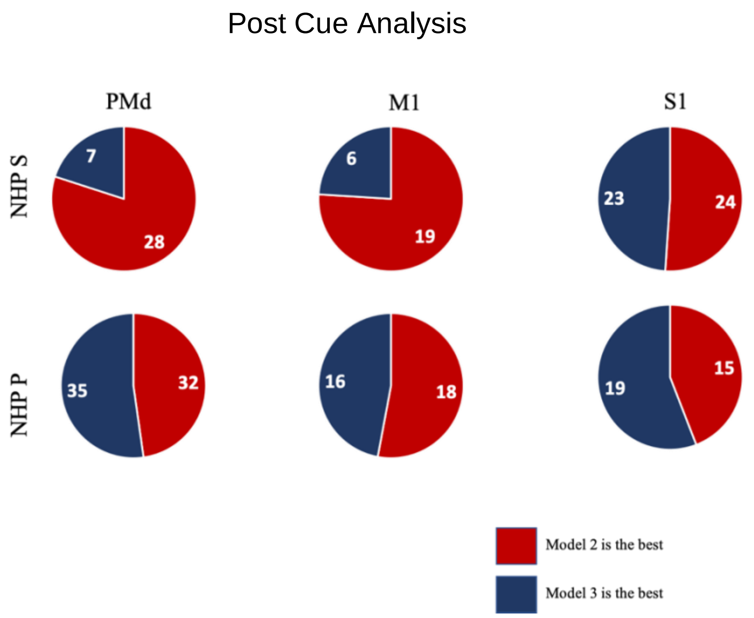

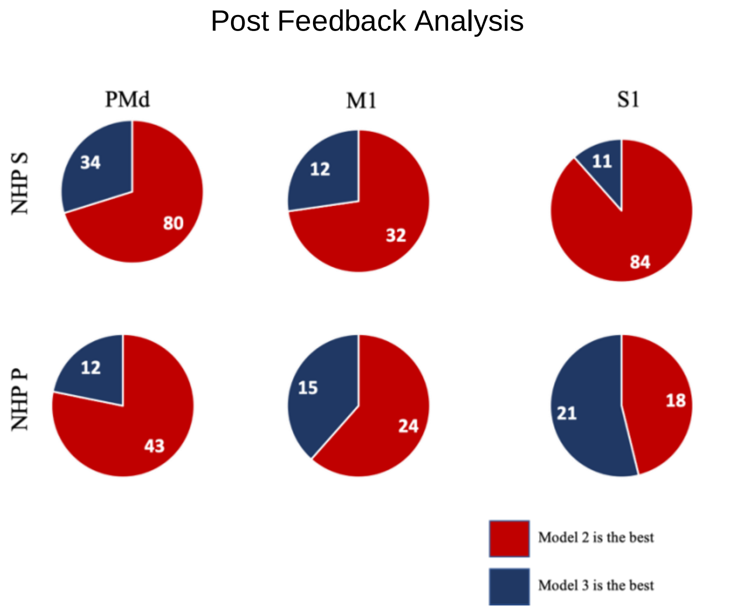

Supplement: Supplementary file 1 — Supplementary Information. [file 41598_2021_3200_MOESM1_ESM.pdf]
